# An audit of Ear, Nose and Throat diseases in a tertiary health institution in South-western Nigeria

**DOI:** 10.11604/pamj.2013.14.1.1092

**Published:** 2013-01-01

**Authors:** Ayotunde James Fasunla, Musa Samdi, Onyekwere George Nwaorgu

**Affiliations:** 1 Department of Otorhinolaryngology, University College Hospital & University of Ibadan, Nigeria

**Keywords:** Audit, awareness, developing country, ENT diseases, presentation, poverty

## Abstract

**Introduction:**

This study is aimed at determining the pattern of ear, nose and throat diseases and their relationship with socio-demographic factors with auditing intent in a tertiary hospital in South-western Nigeria.

**Methods:**

Medical records of patients managed at the Department of Otorhinolaryngology, University College Hospital, Ibadan, Nigeria from 2006 to 2010 were reviewed for all essential clinical data.

**Results:**

There were 2641 (52.8%) males and 2360 (47.2%) females. Two thousand and fifty (41%) patients had age ≤15years old. Sixty three percent of the patients were Christians, 37% were Muslims and less than 1% had other religions. There were more patients in lower occupational classes than those in the upper classes. The average number of patients with ear, nose and throat diseases managed per month was eighty three. Patients with ear diseases were 3136 (62.7%), the nose diseases were 1153 (23.0%), the throat diseases were 479 (9.6%) and head/neck diseases were 233 (4.7%).

**Conclusion:**

This study showed that otitis media, obstructive adenoid, foreign bodies in the ear and throat infections were the common ear, nose, throat disorders seen in patients aged ≤15years whereas, hearing loss, rhinosinusitis and tumors were the common disorders of ear, nose and throat seen in patients aged 16 years and above. Although these disorders are not yet considered to be of public health importance, they contribute significantly to the existing burden of health problems in our environment. Therefore, there is a need for improved public awareness on ear, nose and throat diseases.

## Introduction

Wide varieties of Ear, Nose and throat diseases present to the Otorhinolaryngologist, head and neck surgeons [1]. The pattern of these diseases may vary from community to community or hospital to hospital based on the availability of specialist personnel or facilities for the management of such diseases which are either congenital or acquired in origin. The acquired diseases include infections, inflammatory diseases, neurologic diseases, vascular diseases, trauma, benign and malignant tumors etc. Ear, Nose and throat diseases are serious public health problems with universal distribution affecting all age groups [1, 2]. The knowledge of the ear, nose, throat, head and neck diseases is very important because of the type of morbidities which they cause due to impairment of the inherent physiologic functions that usually take place in the head and neck region. These include problems of hearing, breathing, swallowing, phonation, speech, olfaction, taste, protection of the lower respiratory tract and clearance of secretions. Aesthetic problem of the face and psychological problem may occur in neoplasm and neurologic diseases of the head and neck region. In some situations, these morbidities may lead to social embarrassment, occupational, school and economic losses in the community. The knowledge of these ears, nose and throat diseases can help the administrators and policy makers in the community to make adequate strategic health planning especially in the developing countries, where poverty, ignorance, insufficient personnel and lack of basic health facilities abound. It will also assist the educational sector to define a better medical curriculum for training in Otorhinolaryngology, head and neck surgery in the developing countries. Despite its importance in formulating health care planning and services, there is a dearth in literature on the pattern of ear, nose and throat diseases in developing countries and especially in Nigeria. Therefore, this study was designed to audit ear, nose and throat diseases in a tertiary health institution in Nigeria and to create awareness of its importance in public health.

## Methods

This is a retrospective review of the hospital records of patients that presented and were managed at the Department of Otorhinolaryngology, University College Hospital, Ibadan, Nigeria between 2006 and 2010. This is a health institution which provides tertiary level of health care services and training in South-Western region of Nigeria. The data of the patients collected from the medical registrar included demographic data (age, sex, and religion), Occupational class, diagnosis of presenting problems and surgical treatment offered. The socio-economic status of the patients was based on occupational strata as devised by Famuyiwa et al [3]. Infants & younger children were classified using their parents/caregivers’ occupation and pensioners were classified based on their last job before retirement in this study. The diseases were then grouped based on the location of the complaints into Ear, Nose, Throat and Neck diseases. The results were tabulated and analyzed using descriptive statistics.

## Results

During this study period, five thousand and one new patients were managed for ear, nose and throat diseases at the Department of Otorhinolaryngology, University College Hospital, Ibadan, Nigeria. There were 2641 (52.8%) males and 2360 (47.2%) females with a sex ratio of 1:1 (M: F). The age of the patients ranged from 1day to 86years with a mean and median age of 29years and 24years respectively, standard deviation of 12.23. Adults were considered to be above 15years old and 2050 (41%) patients had age ≤15years old (Table 1). Sixty three percent of the patients were Christians, 37% were Muslims and less than 1% had other religions. Two hundred and thirty nine (4.8%) patients fell into the occupational Class I while Class II, III, IV and V have 786(15.7%), 1233 (24.6%), 1254 (25.1%) and 1489 (29.8%) patients respectively and 83% of the entire patients were urban dwellers. Patients with ear diseases were 3136 (62.7%), the nose diseases were 1153 (23.0%), the throat diseases were 479 (9.6%) and head/neck diseases were 233 (4.7%) and their associations with age are depicted in Table 2. The average number of patients with ear, nose and throat diseases managed per month was eighty three. The four most common ear, nose and throat diseases seen in the patients are shown in Table 3 and Table 4. The average number of patients admitted per year was 367 and out of which an average number of 261 patients underwent surgical treatment in a year.

**Table 1:** Age Distribution of patients in the study population

Age range (Years)	Frequency	Percentage
**0-15**	2050	41.0
**16-30**	1194	23.9
**31-45**	869	17.4
**46-60**	532	10.6
**61-75**	285	5.7
**76-90**	71	1.4
**Total**	5001	100.0

**Table 2:** Relationship of age group with Ear, Nose, throat, Head & Neck diseases

	Age (Years)	Male (n)	Female (n)	Total (n)	Percentage (%)
**Ear diseases**	≤15	763	665	1428	28.5
	>15	908	800	1708	34.1
**Nose diseases**	≤15	158	135	293	5.9
	>15	393	467	860	17.2
**Throat diseases**	≤15	126	149	275	5.5
	>15	131	73	204	4.1
**Head & Neck diseases**	≤15	38	16	54	1.1
	>15	124	55	179	3.6
**Total**		2641	2360	5001	100

**Table 3:** Distribution of various Ear, Nose and Throat diseases of the patients in children (age ≤ 15 years)

Disorders	Diseases	Frequency	Percentage
**Ear**	Otitis media	923	45.0
	FB in the ear	202	9.9
	Hearing loss	153	7.5
	Otitis externa	87	4.2
	Others	63	3.1
**Nose**	Obstructive adenoid	189	9.2
	Rhinosinusitis	34	1.7
	FB in the nose	29	1.4
	Epistaxis	13	0.6
	Others	28	1.4
**Throat/Neck**	Throat infections	162	7.9
	Upper airway obstruction (RRP, FB, laryngo-bronchitis),	57	2.8
	Neck mass (Cysts, tumors)	44	2.1
	Speech disorders	37	1.8
	Others	29	1.4
**Total**		2050	100.0

**Table 4:** Distribution of various Ear, Nose and Throat diseases of the patients in adults (age > 15 years)

Disorders	Diseases	Frequency	Percentage
**Ear**	Hearing loss	1017	34.5
	Suppurative Otitis media	321	10.9
	Otitis externa	179	6.1
	Vertigo (BPPV, Meniere's disease, Labyrinthitis)	103	3.5
	Others	88	3.0
**Nose**	Rhinosinusitis	597	20.2
	Epistaxis	109	3.7
	Sinonasal Tumors	79	2.7
	Facial & nasal trauma	21	0.7
	Others	54	1.8
**Throat/Neck**	Tumors (Nasopharyngeal, oropharyngeal, laryngeal & hypopharyngeal, salivary gland)	207	7.0
	Throat infections	62	2.1
	Upper airway obstruction	51	1.7
	Corrosive & Foreign body ingestion/ aspiration	21	0.7
	Others	42	1.4
**Total**		2951	100.0

## Discussion

This study attempted to determine the pattern of Ear, Nose and Throat diseases of patients seen in a tertiary health institution in Nigeria and afforded the opportunity to have insight into the spectrum of Ear, Nose and Throat diseases in developing countries. An average of 83 new patients with Ear, Nose and Throat diseases visited the hospital every month. This may not be the true representative of patients with Ear, Nose and throat diseases in our environment as most patients treated in our department were referred from primary, secondary or other tertiary health institutions after initial failed treatment by General Medical Practitioners or due to lack of facility to manage the disease condition.

Although the department where this study was conducted was a surgical specialty, only one quarter (21/83) of the patients underwent surgery. The low number of patients that underwent surgical intervention every month may be as a result of the high cost of medical treatment in the hospital which cannot be afforded by an average Nigerian.

The general observation of the patients’ occupational classes showed that there were more patients in lower occupational classes than those in the upper classes. Generally, it is believed that people in upper socio-economic classes are more literate, have healthier lifestyles and behavior than people in lower classes [4]. This might be the reason for this observation in this study. However, lack of time to visit hospitals by carrier builders, business managers, company directors, professionals, civil servants, etc who constitute the upper occupational classes cannot be ruled out as an important factor contributing to this pattern of presentation.

In this study, there was a slight male preponderance and the diseases affected all age groups. About 41% of the patients have age ≤ 15years. The main health problems encountered in the children population in Nigeria were low birth weight, malaria, starvation or malnutrition, diarrhea, and infectious and communicable diseases [5–7]. The ear, nose and throat diseases could either be consequence of the above or may complicate them thereby, adding to the burden of the existing health problems among children in our environment. The focus of the various existing health policies for pediatric population has ignored the significant morbidities that arise from the ear, nose and throat diseases. In this study, about 41% of the pediatric age group had otitis media which constituted the greatest percentage of all the ear, nose and throat diseases diagnosed in them. This is similar to the findings of Kishve et al in India where 31.8% of their study population had otitis media [2]. Ologe et al in Nigeria reported a prevalence of chronic suppurative otitis media among school children in a rural community to be 73 per 1000 pupils [8]. Various similar studies on children have identified otitis media as one of the common ear disorders seen in the group [9, 10]. The increase vulnerability of children to developing Otitis media is a cause of conductive hearing loss in Children [8–10] and commoner among rural dwellers [11]. In this study, otitis media is commoner among patients in occupational classes III and IV. These groups of patients are likely to have nutritional problems and live in overcrowding environments which are important risk factors for the disease. The patients in these classes are likely to delay seeking medical advice due to lack of knowledge about the disease and fund. They are likely to present with associated complications of the disease with increased morbidity.

Children are very inquisitive, eager to explore their environments and probe around the body orifices within the head and neck. Such actions may result in serious medical emergencies. In this study, foreign body in the ear is the second most predominant ear, nose and throat disorder among pediatric patients. The finding conforms to the previous study in the region [12] and the spectra of objects inserted into the ear are similar to those found in other similar studies [13, 14]. The neglect of foreign body within the ear canal may lead to ear infections and impaired hearing. In this study, the methods used for removal of the foreign bodies in the ear were syringing, simple removal with Jobson hornes probe and alligator forceps. The removal of foreign bodies in already traumatized ear due to previous failed attempt(s) at removal from referral center was under sedation.

Obstructive adenoid is the third commonest disorder in the pediatric population in this study. Obstructive adenoid is a common cause of nasal obstruction, rhinorhea and obstructive sleep apnea syndrome [15]. Obstructive sleep apnea syndrome can complicate as obstructive adenoid and it is an absolute indication for removal of an enlarged obstructive adenoid and if neglected, could result in hypoxia, pulmonary hypertension, right ventricular hypertrophy and cor-pulmonale [15, 16].

Hearing loss was a common disorder encountered in this present study. Hearing loss is a significant health problem in developing countries [17, 18]. It is one of the most common birth defects that have a major impact on the lives of the affected infants and their families, with reports that two out of three of the world's hearing impaired people live in developing countries. The reasons for this may include absence of regular screening programs for ear diseases, poverty, malnutrition, ignorance and paucity of accessible healthcare [19, 20]. Infection of the throat is another major disorder encountered in this study. Tonsillitis in children has also been widely reported in literature from different parts of the world [21, 22]. In immunocompromised patients, this could be complicated with tonsillar or neck abscesses as seen in 9 (0.06%) cases of throat infection in this study. Delay in instituting appropriate medical treatment due to ignorance and poverty may also contribute to the development of this complication.

In this study, 41% of the patients had ages between 16 and 45 years whereas less than 8% of the population had ages above 60 years. The level of poverty and joblessness in the country may have contributed to the lower percentage of the elderly patients managed for ear, nose and throat diseases than those patients of the young or middle age groups who belong to the working class. In most health institutions in Nigeria, people still pay directly from their pockets for health services and where there is health insurance, it covers mainly the civil servants in the federal government employments. In addition, this population distribution in this study may actually be a true reflection of the Nigeria population in terms of age where the number or percentage of people in older age group are lower than those in the young or middle age group due to low life expectancy in developing countries.

Fifty eight percent of the adult patients presented with ear disorders of which 34.5% were cases of hearing loss. Hearing apparatus is an important organ in the body that is very useful in day-to-day communication with the outside world, smooth performance of activities and social development. When this organ is diseased, it can result in impairment of function (hearing and balance). Hearing loss in an adult can be caused by aging process (presbyacusis), infections of the ears, drugs, trauma etc [23, 24]. Rehabilitation of hearing after eradication of the cause of hearing loss is important for an improved quality of life.

In this study, Rhinosinusitis is the second most predominant ear, nose and throat disorder among adult patients. It is the fifth most common diagnosis for which an antibiotic is prescribed and accounted for 21% of all adult antibiotic prescriptions [25]. It is a common clinical condition which if left unattended to could result in various degrees of both morbidity and mortality [26, 27]. It could be infectious or non-infectious in etiology. The osteomeatal complex (anterior ethmoid-middle meatal complex) is a key area in the pathogenesis of rhinosinusitis and blockage of the ostia of the paranasal sinuses is an important factor in the chronicity of the disease. The common predisposing factors include viral upper respiratory tract infection, acute exacerbation of allergic rhinitis, septal deviation, dental infection or tooth extraction, foreign body or trauma to the sinuses etc [25–27]. Re-aeration of the obstructed paranasal sinuses, reduction of nasal secretion and obstructions are important aspects of the management of the disease.

Tumors of the nose, throat and neck accounted for 8.7% of the adult ear, nose and throat disorders in this study. The variety of these tumors seen in this study is similar to what had been previously reported from this environment [28]. However, late stage disease presentation, due to factors such as ignorance, poverty, and initial unorthodox medical practices, is a common finding in most of the patients [29]. Tumors of the larynx, oropharynx and hypopharynx may result in upper airway obstruction necessitating use of tracheostomy tube to safe life.

Accidental aspiration or ingestion of foreign body like denture is seen more in the elderly patients in this study. This may be related to aging process such as decreased connective tissue bulk and decreased pharyngeal and esophageal muscle tones. Persistence of some age related neurological disorders may result in swallowing disorder which can predispose patient to aspiration [28].

## Conclusion

This study showed that otitis media, obstructive adenoid, foreign bodies in the ear and throat infections are the common ear, nose, throat disorders seen in patients aged 15years and less whereas, hearing loss, rhinosinusitis and tumors are the common disorders of ear, nose and throat seen in patients aged 16 years and above. Although these disorders are not yet considered to be of public health importance, they contribute significantly to the existing burden of health problems in our environment. The possibility of low level of public enlightenment on ear, nose and throat disorders, financial constraint, and lack of time or negligence of health cannot be ruled out as important factors contributing to pattern of presentation. Therefore, there is a need for increased awareness of the people in developing countries especially in Nigeria through social campaigns and health education aimed at providing quality ear, nose and throat health care services.
